# Scalable estimator of the diversity for de novo molecular generation resulting in a more robust QM dataset (OD9) and a more efficient molecular optimization

**DOI:** 10.1186/s13321-021-00554-8

**Published:** 2021-10-02

**Authors:** Jules Leguy, Marta Glavatskikh, Thomas Cauchy, Benoit Da Mota

**Affiliations:** 1 Univ Angers, LERIA, SFR MATHSTIC, 49000 Angers, France; 2 Univ Angers, CNRS, MOLTECH-ANJOU, SFR MATRIX, 49000 Angers, France

**Keywords:** Chemical space exploration, Organic chemistry, Quantum chemistry dataset

## Abstract

Chemical diversity is one of the key term when dealing with machine learning and molecular generation. This is particularly true for quantum chemical datasets. The composition of which should be done meticulously since the calculation is highly time demanding. Previously we have seen that the most known quantum chemical dataset QM9 lacks chemical diversity. As a consequence, ML models trained on QM9 showed generalizability shortcomings. In this paper we would like to present (i) a fast and generic method to evaluate chemical diversity, (ii) a new quantum chemical dataset of 435k molecules, OD9, that includes QM9 and new molecules generated with a diversity objective, (iii) an analysis of the diversity impact on unconstrained and goal-directed molecular generation on the example of QED optimization. Our innovative approach makes it possible to individually estimate the impact of a solution to the diversity of a set, allowing for effective incremental evaluation. In the first application, we will see how the diversity constraint allows us to generate more than a million of molecules that would efficiently complete the reference datasets. The compounds were calculated with DFT thanks to a collaborative effort through the QuChemPedIA@home BOINC project. With regard to goal-directed molecular generation, getting a high QED score is not complicated, but adding a little diversity can cut the number of calls to the evaluation function by a factor of ten

**Supplementary Information:**

The online version contains supplementary material available at 10.1186/s13321-021-00554-8.

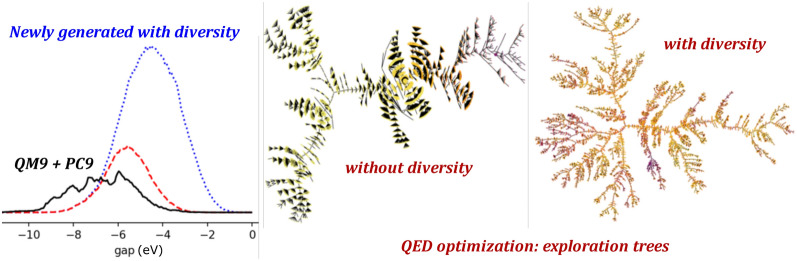

## Introduction

Many applications in the field of molecular chemistry rely on specific electronic properties. In order to evaluate these properties precisely, quantum chemistry calculations are necessary. But these calculations are costly in terms of time and computing resources. This can slow down the discovery of new compounds. One of the great hopes of using machine learning (ML) methods in chemistry is to be able to reduce the amount of quantum chemistry calculations or even bypass them [1]. The cost of calculations with ML methods is much lower. Being able to estimate the interest of a molecule by ML methods would therefore greatly accelerate the discovery of new materials [2]. It has also been shown recently for solar cell materials [3–7], other light-matter based devices [8, 9] and reviewed for a wide range of energy materials [10].

Supervised ML methods greatly depend on the size and quality of the dataset for good performances in generalization. In a previous study we have shown that the most widely used quantum chemistry dataset for small organic molecules, QM9 [11], lacked chemical diversity [12]. A model trained on QM9 could be quite accurate for classical organic chemistry. However it would propose very bad estimations for under-represented chemical functions such as peroxide and derivatives, diaryl ethers and diaryl amines, etc. [12]. The QM9 dataset includes one third (134k) of the molecules with up to nine heavy atoms (C, N, O and F) of the GDB database, the chemical space enumeration attempts by Reymond et al. [13–15]. Furthermore, this chemical space enumeration is not exhaustive. The goal of the GDB is to serve as a catalog of supposedly stable and realistic molecules for virtual screening. Constrained molecules, aminals, acyl fluorides and other reactive compounds have been discarded during the creation of the GDB since a full combinatorial approach would lead to several million molecules [14]. This impressive sampling of the chemical space was indeed not designed to represent the exploration playgrounds of molecular materials chemistry that looks for peculiar compounds with uncommon (electronic) properties. To study the chemical diversity of QM9, we have proposed the PC9 dataset a subset of the PubChemQC that could be compared to QM9 [12, 16]. Very recent studies comparing QM9 and our PC9 dataset indicate indeed better performances when trained on a more diversified dataset [17–19].

We have also recently published a generator, EvoMol, based on a genetic algorithm [20]. On different problems, it has shown very good performances in optimization. But the study of the solutions shows that good candidates are very similar. This is the consequence of our algorithm, which intensifies around the best solutions. ML-based generators see also their solutions biased, by the datasets or by some specific issues. We can cite for example GANs which are prone to *mode collapse*. The generator rotates through few different solutions and mechanisms should be included to prevent this. A similar problem occurs with reinforcement learning (RL), where a learned policy without introduction of randomness always leads to one and only one molecule (policy collapse). Beyond the addition of randomness, several RL-based generators included other mechanisms to increase diversity [21–23]. These solutions are quite specific to the proposed generator or to the generation method. We believe that the inclusion of a diversity criterion can improve the interest of the solutions proposed by any generator. During the course of this study, Kwon et al. published an article where they add a criterion based on the Tanimoto similarity on fingerprints into their evolutionary algorithm [24]. This article confirms our opinion because MolFinder maintains great optimization performances in a reference benchmark despite this additional diversity criterion.

Therefore the aim of our study here is to propose a new method to calculate the contribution of a compound to the chemical diversity of a set. In a first application, we will see how this method allows us to generate molecules that would efficiently complete the reference datasets. Then we will study the impact of this diversity constraint on the set of solutions of a classical problem such as a QED optimization.

The concept of chemical diversity is not clearly defined. On which criteria and with which metrics should diversity be measured? In the field of de novo molecular generation, the term diversity usually refers to external diversity, where the generated compounds are compared to a reference dataset. Some benchmarks propose to measure external diversity using dedicated distribution learning tasks [25–27]. The metrics commonly used are mathematical tools to compare distributions (e.g. KL divergence [28]) or curves (e.g. Fréchet distances [29]). The descriptors used can be of different nature, such as physicochemical descriptors (molecular weight, number of aromatic rings, etc.), structural features [26] (BRICS fragments [30] and Bemis-Murcko scaffolds [31]), or internal descriptors of the ChemNet neural network [32]. It is also possible to define a distance, often the Tanimoto distance [33], between two fingerprints, often ECFP4 [34], and thus calculate the average distance to a set of reference points [35].

In our case, this external diversity is not appropriate. We do not want to refer to another set of data but to measure the internal chemical diversity. A molecular generator that includes an objective of diversity has been proposed by Nigam et al. [36]. Similar molecules are penalized by a neural network discriminator in order to kill long-surviving molecules and thus promote the exploration process. The chemical diversity has been more often studied by the mean of scaffolds analysis [37–43]. Another common metric used to measure diversity is the mean distance between the molecules using Tanimoto distance [33] on fingerprints. Benhenda et al. [27] also proposed to use nearest neighbors, entropy and the Wasserstein distance. The aim of our work here is not to compare the different approaches to diversity but to select one that is effective in the context of molecular generation. In order for a population-based molecule generator to integrate it as an objective, it is necessary to choose a metric allowing to determine quickly the contribution of each molecule to the diversity of a set.

We present in this paper a fast and chemically meaningful way to compute the internal diversity of a dataset. We propose two experiments to demonstrate the interest of this approach. Firstly, we optimize only this objective in order to generate a more diversified dataset of molecules up to nine heavy atoms among C, O, N and F. The 435k compounds of this dataset, called OD9, were calculated with DFT thanks to a collaborative effort through the QuChemPedIA@home BOINC project. We will present in detail the diversity of the newly generated compounds (250k) compared to the reference datasets (QM9 and PC9). Secondly, we integrate the internal diversity objective with the QED [44], to show the impact on the generated solutions and how the exploration of the chemical space is impacted.

## Methods

### Quantification of the diversity, descriptors and implementation

As stated in the introduction, the objective of diversity must be calculated for each molecule and account for a contribution of the chemical diversity in the current dataset. For this purpose, we chose the Shannon entropy and we have selected several candidate descriptors : scaffolds, functional groups and shingles. The Jaynes’ maximum entropy principle can be stated as follows: a distribution with the maximum entropy implies minimal assumptions about the true distribution of data [45, 46]. One can easily see the interest of this principle to generate a diversified dataset when considering the chemical constraints, the distribution of the descriptors is neither equiprobable nor known. In addition, knowing that some optimization problems are solved more efficiently if the portfolio of solutions is diversified [47], we also believe that this approach could be useful for all population-based molecular generators.

*Shannon entropy* The entropy of a dataset *X*, described by *n* binary descriptors for which the proportion of the ith descriptor in *X* is denoted $P_i(X)$, is defined by Eq. 1.

1 $$\begin{aligned} H(X) = \sum \limits _{i=1}^n -P_i(X) \log P_i(X) \end{aligned}$$

Each term in the summation is 0 for $P_i(X) = 0$ or $P_i(X) = 1$, and reaches its maximum value for $P_i(X) = e^{-1}$. Adding a rare descriptor contributes more to the entropy of a dataset than a common descriptor. Very common descriptors contribute very little to the entropy. Thus, to maximize *H*(*X*), the distribution of all descriptors must be as balanced as possible. Equation 1 only takes into account the on-bits. To have the complete entropy considering this equation, it would be necessary to integrate for each vector of descriptor the complementary vector (where the 0’s are 1’s and vice versa). For all the remainder of this section, we consider only the on-bits part for two main reasons. First, we will try to quickly evaluate the diversity and this simplification divides by 2 the amount of computation. Secondly, the descriptor vectors are in general very sparse and this simplification has no impact. One could even find that favoring a larger amount of off-bits is an advantage. When the proportion of on-bits exceeds $e^{-1}$, the investigator will have to chose between this imbalance or a classical equilibrium (a proportion at 0.5). In that case, it will be necessary to integrate the complementary vectors to use our estimator in its complete version of the entropy.

In a naive way, it is possible to calculate the entropy of the dataset without a molecule *m* in order to evaluate the contribution $\Delta _{r}(m,X)$ of *m* in *X* with Eq. 2.

2 $$\begin{aligned} \Delta _{r}(m, X) = H(X\setminus \lbrace {m}\rbrace ) - H(X) \end{aligned}$$

The contribution for adding a molecule can be calculated in the same way.

3 $$\begin{aligned} \Delta _{a}(m, X) = H(X\cup \lbrace {m}\rbrace ) - H(X) \end{aligned}$$

Thus, it is possible to transform a global problem where the aim is to maximize diversity into a problem of optimizing individuals. It is possible to remove a molecule that decreases or contributes only a little to the diversity or to choose a molecule that, on the contrary, increases it. Above all, it is possible to rank them.

*Computational efficiency* Calculating the contribution of a molecule with Eqs. 2 and 3 is very expensive. With a dataset of several thousands molecules and a rich chemical diversity (several different descriptors), this equation would be a limiting point in terms of computing time. However, we have chosen descriptors which for each molecule are in limited number, i.e. one scaffold and less than ten IFGs. It is thus possible to no longer consider molecules, but the space of descriptors (denoted $^*$). The key point to speed up the computation is to consider that the size of the dataset is constant during the entire optimization process. Even when starting with a single molecule, it will always be the final size of the dataset that will be used at any time. This approximation (denoted $\Delta '$), allows to reuse a very large amount of calculation.

The entropy of a descriptor $D_i$, with $C_i(X)$ the number of occurrences of the descriptor in the dataset, and |*X*| the size of the dataset is defined as in Eq. 4.

4 $$\begin{aligned} H^*(D_i, X) = - \frac{C_i(X)}{|X|} \log \frac{C_i(X)}{|X|} \end{aligned}$$

We can define the entropy variation for a descriptor $D_i$ by removing a molecule *m* containing or not this descriptor. As we will see later, we will add as many molecules at each step of the optimization as we remove. In the space of descriptors, this is equivalent to removing *m* and adding an empty molecule $\emptyset$, i.e. without descriptor, to obtain a dataset of the same size. In this case, if the molecule does not contain the descriptor $D_i$, the variation of entropy is 0 (see Eq. 5).

5 $$\begin{aligned} \begin{aligned} \delta ^*_{r}(D_i, m, X) =&H^*(D_i, (X \setminus \lbrace {m}\rbrace ) \cup \lbrace \emptyset \rbrace ) \\&-H^*(D_i, X) \end{aligned} \end{aligned}$$

Thus, the contribution of a molecule *m* is calculated by only considering the few descriptors involved in *m* (see Eq. 6).

6 $$\begin{aligned} \Delta '_{r}(m, X) = \sum \limits _{D_i \in m} \delta ^*_{r}(D_i, m, X) \end{aligned}$$

This equation allows to sort the molecules by contribution to the total entropy of the dataset. In an algorithm, when a molecule must be removed, this equation should be used. In a very comparable way, it is possible to evaluate the variation in entropy caused by the addition of a molecule, denoted $\Delta '_{a}(m, X)$, since it is the opposite operation (see Eqs. 7 and 8).

7 $$\begin{aligned} \begin{aligned} \delta ^*_{a}(D_i, m, X) =&H^*(D_i, (X \setminus \lbrace {\emptyset }\rbrace ) \cup \lbrace m \rbrace ) \\&-H^*(D_i, X) \end{aligned} \end{aligned}$$

8 $$\begin{aligned} \Delta '_{a}(m, X) = \sum \limits _{D_i \in m} \delta ^*_{a}(D_i, m, X) \end{aligned}$$

This equation is used to rank the compounds proposed by a molecular generator. If the set of solutions is of limited size, this value alone is not sufficient since the decision to add a molecule must take into account the contribution of the molecule it will replace and these two molecules may share common descriptors. Let $m_r$ be the molecule to be removed and $m_a$ the molecule to be added, then $m_a \setminus m_r$ denote the molecule $m_a$ without the descriptors of $m_r$. It is then possible to define the entropy variation of a substitution $\Delta '_s(m_r, m_a, X)$ (see Eq. 9).

9 $$\begin{aligned} \begin{aligned} \Delta '_{s}(m_r, m_a, X) =&\Delta '_{r}(m_r \setminus m_a, X) \\+ & {} \Delta '_{a}(m_a \setminus m_r, X) \end{aligned} \end{aligned}$$

if the value of $\Delta '_s$ is greater than or equal to 0, the substitution is considered as an improving one. With this approach it is possible to update only the scores of molecules that share one or more descriptors with $m_r$ ou $m_a$. Moreover, depending on the size of the dataset to be considered, the update can take place only after a certain number of substitutions.

To summarize, our entropy calculation is an approximation, performed in the dual space of the descriptors. The approximation comes from two main reasons. First, the size of the population is considered as constant to optimize the caching of results, which saves a lot of calculation. Secondly, in our implementation we operate by batch and we do not consider the interactions between the molecules that are added and removed. The entropy gain is considered only with the dataset before the beginning of the batch treatment as reference. These approximations are reasonable and necessary for the feasibility of practical applications.

*Scaffolds* One of the most commonly used molecular descriptor to assess the diversity is the molecular framework or scaffold [37–43]. Originally defined by Bemis and Murcko for drug design, the molecular graph does not take into account side chains to focus on cycles and their linkers [31]. In fact, different levels of abstraction or scaffold hierarchy have been used in some scaffolds analysis on the PubChem for example [42]. In this article, we will designate by the term scaffold, the framework returned by RDKit [48]. It still takes into account the unsaturations and the atom type but neglect the side chains.

*Generic Scaffolds.* In our previous study, we have seen that the diversity in the chemistry of the side chains and of the acyclic compounds was quite different between QM9 and PC9 [12]. Therefore, we will also use an alternative approach to generate what we will call here generic scaffolds. All heteroatoms are transformed as carbons and all bonds are considered as single bonds. The benzene and the cyclohexane have the same generic scaffolds but the toluene and the benzene will not anymore.

We have integrated either the scaffolds or the generic scaffolds into our objective function to improve the topological diversity of the dataset.

*Functional groups: CheckMol and IFGs* In our previous study concerning the diversity, we have also seen that functional groups underrepresented in a training dataset could lead to huge errors in machine learning based predictions. The role of functional groups in the properties of an organic compound even shapes the way of teaching organic chemistry. There is two main solutions for the automatic classification inside an established chemical ontology, the CheckMol and ClassyFire programs [49, 50]. However, our unconstrained nature of generating molecules will leads to uncharted or neglected new sets of connected atoms i.e. new functional groups. Therefore, we have chosen to use the automatic approach of identifying functional groups proposed by Peter Ertl [51]. It is centered on heteroatoms and their surroundings (atoms and bonds). It will merge connected surroundings to form new identified functional groups (IFGs). We have used the IFG detection program as implemented by Guillaume Godin and Richard Hall for the RDKit package [52].

*Shingles* Finally, we have used an automatic and unbiased approach of defining chemical moieties called shingles. They are subgraphs centered around each atoms. Depending on the cut-off radius, noted r, they can capture the chemical environment up to 3 bonds away (r$=$3). We have used the shingles detection program as proposed by the group of Jean-Louis Reymond for the calculation of the CLscore [53, 54] which also relies on the RDKit package [52].

*Combining several categories of descriptors* The contribution of each category of descriptors are computed separately, scaffolds and IFGs for instance, in order to be able to weight each one in an objective function (see Eq. 10). It would be easy to add other descriptors and weight them in this way.

10 $$\begin{aligned} \Delta^{\prime}_{s}(m_r,m_a,X) &= \omega _{\text{ifg}} \Delta^{\prime}_{s_{\rm ifg}}(m_r,m_a,X) \\ & \quad +\omega _{\text{scaf.}} \Delta^{\prime}_{s_{\rm scaf.}}(m_r,m_a,X)\\ & \quad + \cdots \end{aligned}$$

Between the topological diversity measured by the scaffolds and the functional groups diversity measured by the IFGs, we think that an objective function that combines both can deal with classical chemistry and also unstable molecules. It is this combination that we have chosen to generate unconstrained diversity (cf. section "Case 1: unconstrained molecular generation"). On the other hand, when optimizing the QED property, we studied CheckMol, IFGs and shingles separately to observe the impact of the choice of descriptors (cf. section "Case 2: goal-directed molecular generation"). One would expect this choice to have an impact on the balance between diversity and drug-likeness. This study is also not exhaustive and focuses on three structural descriptors that we think to be relevant for the diversity of chemical functional groups. It illustrates how the choice of descriptors is problem dependent.

The method we propose is not dependent of a particular generator. It allows to evaluate the diversity contribution of a compound in a dataset at a given time of any generation process. In this work we evaluate only descriptors directly related to the structural diversity of molecules. However, it is possible to integrate descriptors less directly related to the structure, such as individual bits of fingerprints, or even continuous descriptors completely unrelated to the structure. For the latter case, it would be necessary to define bins for the values of the continuous variable, then to perform a one-hot encoding. A generator able to optimize complex tasks, will also be able to optimize a diversity like any other objective, even if defined on non-structural descriptors.

*Molecular generator* In order to realize our experiments, we have implemented the diversity objective in EvoMol [20], which has all required characteristics. First of all, it is an evolutionary algorithm that optimizes a population of molecules by eliminating those furthest from the target and replacing them with improvers obtained by mutation of the best individuals in the population. As stated before, the size of the dataset is constant and operations are done in batches. Then, the flexibility of this generator allows to quickly adapt it while its interpretability allows to visualize the impact of the diversity on the chemical space exploration (see section "Case 2 : goal-directed molecular generation"). Finally, due to its very unconstrained nature, it is very efficient in optimization and generates molecules that are sometimes unrealistic and not very stable. In our case, it is a useful property to increase the chemical diversity of reference datasets as we will demonstrate in section Case 1: unconstrained molecular generation.

### Experiments

We are conducting two experiments. The first one consists in generating as much internal diversity as possible without any other objective and to analyze the resulting datasets. The second aims to demonstrate the benefits of including diversity as part of the optimization of a joint objective in order to avoid intensification around a single solution.

#### Case 1: unconstrained molecular generation

In this large-scale experiment, we aim to maximize chemical diversity using our methodology presented above. The newly generated compounds respect all the constraints of QM9 and PC9, i.e. maximum nine heavy atoms among C, N, O and F. Since our objective is an optimization of diversity, we have named the resulting dataset OD9. OD9_0 will refer to $\mathrm {QM9} \cup \mathrm {PC9}$, and OD9_1 the newly generated compounds. More than one million new compounds have been generated and all OD9 molecules have been calculated in DFT with the same calculation parameters and a strict quality control. Due to high failure rate of the DFT and the quality control with the new compounds, the whole chain was executed 6 times with slightly different parameters. To guarantee the uniqueness of the generated compounds, already known molecules were removed after each of the 6 iterations. For the first two runs, the descriptors used were IFGs and scaffolds, while in the following runs we used IFGs and generic scaffolds. For the first four runs, the starting dataset was $\mathrm {QM9} \cup \mathrm {PC9}$, while for the two last runs we only started from methane. Since the differences in parameters did not result in significant changes, we made the union of the results of the 6 executions in OD9_1 to analyze them together. The entire workflow is presented below.

**Step 0 (generation)** The first step consists in generating with EvoMol about 210k molecules, i.e. approximately the size of the union of PC9 and QM9 without duplicates. EvoMol was used with primary actions only (append, substitute and remove atom, change bond) with the sole objective of maximizing diversity of equal weight between IFGs and Scaffolds (${\omega}_{\text{ifg}} = {\omega}_\text{{scaf.}}$). The batch size was 100 and each mutation consists in applying exactly 3 random actions. When an improver was found, the molecule was validated with RDKit molecular mechanics (MMFF). EvoMol was stopped after a few hours, when the diversity no longer increased significantly. We thus obtain a list of potential SMILES candidates from which we removed molecules already proposed in another execution or already present in PC9 or QM9.

**Step 1 (submissions)** At this stage of the workflow, there remain all the unique molecules generated for which molecular mechanics has been able to produce a starting geometry with the same canonical SMILES than the generated molecular graph. Then, all these molecules were submitted to the BOINC server to be calculated in DFT with NWchem [55] using the B3LYP functional and the 3-21G basis set. Details of the functioning and particularities of BOINC [56] are described in a dedicated paragraph after the description of the full workflow.

**Step 2 (quality check)** At this stage, the remaining molecules must have two calculations that have converged to the same stationary point. The output files are therefore present and have an indication of successful termination. In addition, we check that there are no NaN in values of interest and that the final geometry can be discretized to obtain a canonical SMILES. Then, the molecules that are dissociated are filtered out, as well as those who have converged to a point far from a minimum with large negative vibrational frequencies ($< -20\ cm^{-1}$).

**Step 3 (stable and unique)** This stage consists in checking that the SMILES of the molecule has remained identical before and after DFT. In the rest of the article, when we talk about stability, it will refer to this stability to the DFT calculation. A final uniqueness check is performed without taking into account the stereochemical information.

*Berkeley Open Infrastructure for Network Computing (BOINC)* We have set up an instance of BOINC, named QuChemPedIA@home, to allow us to distribute our numerous calculations on our machines and on the computers of the volunteers. Inputs generation for NWchem was automated and a native wrapper for Linux operating systems was developed. The contribution of other operating systems is allowed by the use of a virtual machine and the official wrapper with BOINC and Virtualbox. So much heterogeneity implies many calculation errors, but the system is designed for this and failed tasks will be submitted a number of times on different hosts before being declared in error. As volunteers are not paid, the incentive to calculate is managed with credits and rankings. This implies, that it is necessary to take some measures in order to avoid cheating and erroneous results. For this, a quorum system is implemented which implies that two volunteers must find results close enough to obtain the credits. Our quorum procedure is a rather loose comparison of total energy and nuclear repulsion energy. Despite those thresholds, many molecules have failed to reach a quorum, not due to cheating. Although the different calculations start from the same geometry calculated in molecular mechanics, the results can vary enormously. On the one hand, the execution of NWchem is not deterministic on two different machines. On the other hand, the molecules proposed by our generator are very unstable and often reorganize during DFT. These preliminary observations will be confirmed at the final analysis of the dataset (see section "Case 1: unconstrained molecular generation"). Still active, this BOINC project allow us to produce more reproducible results for millions of molecules.

#### Case 2: goal-directed molecular generation

In this second experiment, the goal is to jointly optimize an objective, in this case the QED, and the diversity. The QED is a metric evaluating the drug-likeness based on the similarity of the distributions of a set of properties with known drugs [44]. As there are many ways to get a high QED score, incorporating diversity allows for quite different solutions. The second effect that we would like to highlight is less obvious. Without diversity, we have observed that EvoMol can intensify around the best current solution without finding any improvement in the end and will be forced to abandon this path. With the right amount of diversity, over-explored branches should be more quickly abandoned in favor of other paths, leading more quickly to good solutions.

As in the EvoMol paper [20], we conduct our space exploration on molecules containing C, N, O, F, P, S, Cl or Br atoms, with molecules up to 38 heavy atoms. The size of the population was set to 1000 where 10 individuals are replaced at each step, during 800 steps or until convergence. The other parameters of EvoMol were set to default. The weight of the QED was set to 1 and the weight of diversity has taken different values (0, 0.1, 1, 10, 100 and 1000). Different descriptors for diversity have also been tested: CheckMol functional groups, IFGs and shingles (radius 1). We have not optimized the diversity of scaffolds, because with molecules up to 38 heavy atoms, their number is colossal. Diversity can only be relevant if the size of the population is (much) larger than the number of descriptors. For all these parameters 10 runs are performed and we studied the diversity in terms of the number of descriptors, the speed of convergence of the best solution according to the number of calls to the objective function and the effect on the exploration tree.

## Results and discussion

### Case 1: unconstrained molecular generation

#### Diversity analysis on scaffolds and IFGs

As a starting dataset we have gathered all the SMILES of the union of QM9 and PC9. All these molecules were then recalculated twice with the BOINC project. Following the workflow presented in section Case 1: unconstrained molecular generation, some compounds are discarded if they are radicals or duplicated, if they are too far from a global minimum, if their 3D structure does not allow to find a canonical SMILES or if their final SMILES is different from the original SMILES. At the end of this process, 122,227 QM9 compounds and 77,790 PC9 compounds were retained. The cleaning of radicals and duplicates has had a significant impact on PC9. The union of these two sets is noted OD9_0 to indicate that this is our starting point. After step 3, this union contains only 184,158 molecules after elimination of the duplicates between QM9 and PC9. The detail of the evolution of the number of compounds at each step for all datasets is given in Table 1 of Additional file 1.

The chemical diversity in terms of generic scaffolds, scaffolds and IFG is reported in Table 1. Comparing QM9 and PC9, we can confirm our previous analysis [12]. There is less topological diversity (generic scaffolds), alkyl diversity (acyclic scaffolds) and heteroatoms diversity (distinct IFG) in QM9 than in PC9. There is however more diversity in QM9 when we consider the unsaturations (see the distinct scaffolds column) since the double bonds were placed with a combinatorial approach when in PC9, which is a subset of PubChemQC [16], there is predominantly aromatic compounds. Considering OD9_0 after curation (step 3), we could note a few tens of thousands distinct scaffolds or IFG. So, we were quite confident in their ability to serve as meaningful descriptors during the de novo molecular generation process.

**Table 1:** Scaffolds and IFG statistics for the datasets at different stages of the workflow

Dataset	Size	Distinct generic scaffolds	Distinct scaffolds	Acyclic graphs	Distinct IFG
QM9 step 3	122,227	1964	14,060	12,615	6981
PC9 step 3	77,790	2772	6566	31,542	13,887
OD9_0 step 3	184,158	3798	18,850	40,103	20,075
OD9_1 step 1	1,023,624	9163	460,978	28,725	461,247
OD9_1 step 2	854,059	108,832	334,256	66,078	428,136
OD9_1 step 3	250,874	4858	88,094	15,956	124,396
OD9 step 1	1,276,171	12,929	480,464	90,965	482,009
OD9 step 2	1,088,773	109,573	351,845	122,771	446,367
OD9 step 3	435,032	6776	104,529	56,059	141,090

OD9_1 is the set of SMILES generated with an objective of diversity and that do not belong to OD9_0. This represents slightly more than one million SMILES at step 1, i.e. before DFT calculation. Looking at the lines corresponding to OD9_1 in Table 1, it is possible to see that our objective function for diversity has reached its goal. The expansion in terms of scaffolds and IFGs is impressive. The OD9_1 set at step 1 contains more different generic scaffolds, scaffolds and IFGs than the union of PC9 and QM9. Most of these new scaffolds or IFGs appear only once which can be a problem for machine learning (see a more detailed version with Unique columns in Table 2 of Additional file 1). We can notice that the combination of scaffolds and IFGs in our objective function did not reward EvoMol for exploring the acyclic chemistry.

When we compare in Table 1, the OD9_1 at steps 1 and 2, we can see that the proposed 9163 generic scaffolds converged after DFT into 108,832 generic scaffolds, indicating spacial and chemical rearrangements. Indeed, only 250,874 SMILES remained at step 3, identical to the ones at step 1. The generation of chemical diversity pushes the generator to explore structures that do not write well in the form of discretized SMILES.

**Fig. 1:**
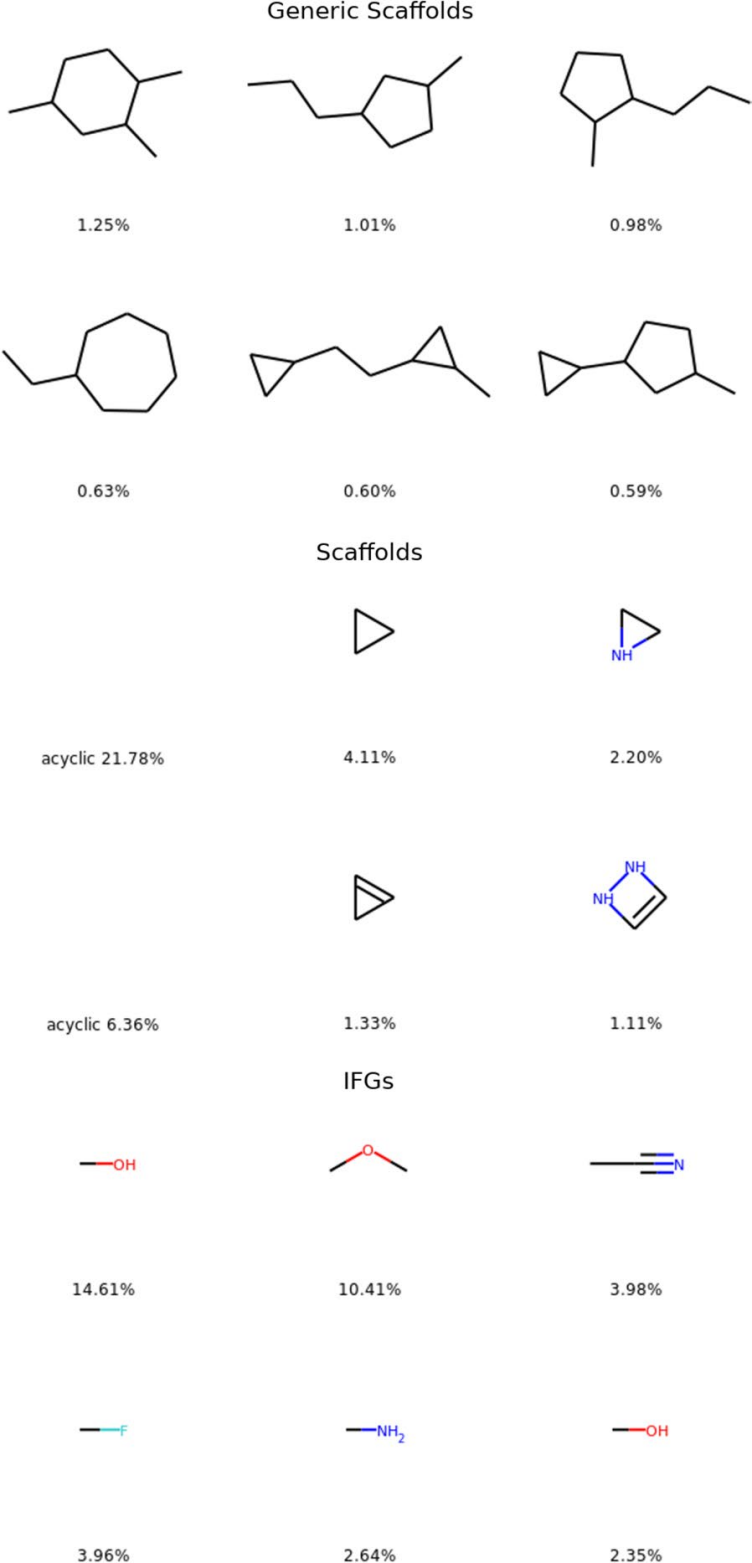
Top 3 Generic scaffolds, scaffolds and IFGs for OD9_0 (up) and OD9_1 step 3 (down)

In Fig. 1, the top three for each descriptor are represented along with the percentage of molecules presenting this descriptor. This figure confirms the success of this experience of generating diversity. Keep in mind that OD9_1 has been build to generate compounds that would add diversity to OD9_0. Looking at the generic scaffolds, we can see for OD9_0 that six and five members rings are in the top 3, when in OD9_1 seven members rings and separated 3 members rings are found. For scaffolds, the overall acyclic category is always the first but with a drastic change in terms of ratios (21.78% compared to 6.36%). To complete the top 3 in OD9_0 we find small saturated rings for unsaturated ones in OD9_1. Finally, the IFGs highlight the change in chemistry in OD9_1 with much less in proportion of hydroxyl groups, dialkyl ethers and nitrile and a much more balanced distribution.

**Fig. 2:**
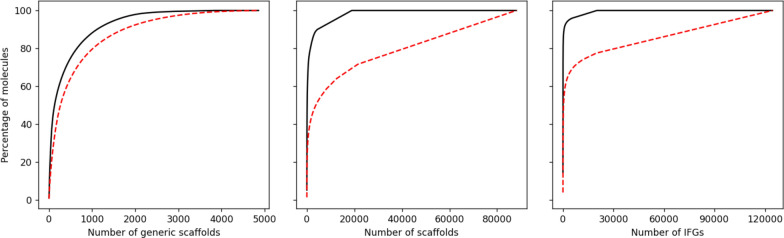
Cumulative plots of the generic scaffolds, the scaffolds and the IFGs for the OD9_0 (QM9UPC9 in black straight lines) and for the newly generated OD9_1 step 3 compounds (in red dashed lines)

For all those descriptors the percentages of occurrence are much lower in OD9_1 than for OD9_0. To better visualize this distribution, we have calculated their cumulative plots (see Fig. 2). We can thus observe that the distributions in the generated compounds (OD9_1) are more balanced with curves closer to a linear growth especially for the scaffolds and the IFGs. With nine heavy atoms we have generated more generic scaffolds in OD9_1 step 3 (4858 compared to 3798 as shown in Table 1) but there is a large overlap between the generic scaffolds of the two datasets since their sum correspond to 6776 distinct generic scaffolds. We have probably computed all reasonable generic scaffolds that pass the DFT. This is not the case for the other two descriptors.

#### Analysis on non optimized scores or descriptors

We can also compare the chemical diversity between OD9_0 and OD9_1 through properties that we did not directly optimize. We have selected two indices of synthesizability and some electronic properties to evaluate as distributions the diversity of those two datasets.

**Fig. 3:**
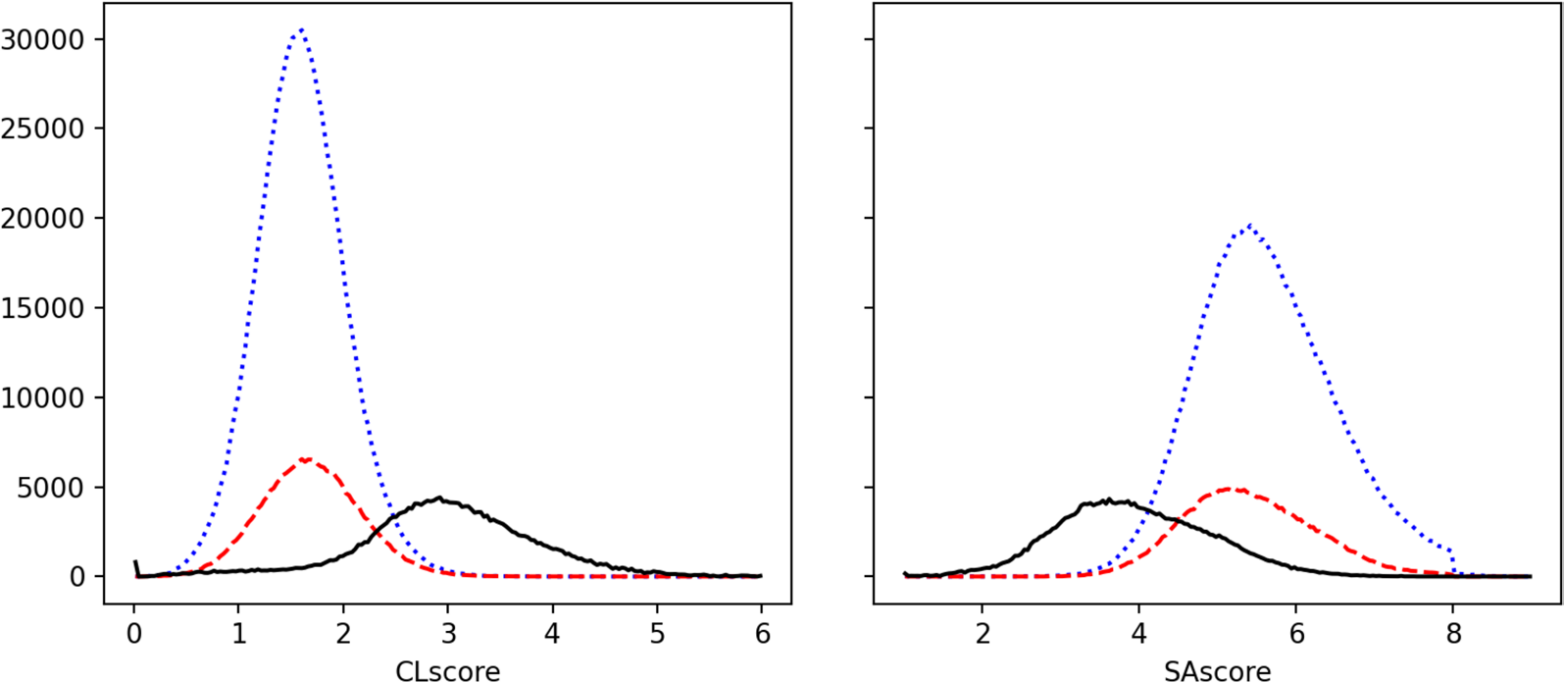
Distributions of the CLscore and SAscore. The black straight lines represent the OD9_0 dataset (QM9UPC9). The new generated compounds OD9_1 are represented by the blue dotted lines for the step 1 or the red dashed lines for the step 3 of the workflow

The CLscore and SAscore indices have been designed to estimate the synthesizability or complexity of a molecule mainly by comparing it with the most common fragments of respectively the ChemBL and the PubChem [54, 57]. A high CLscore is expected for a molecule mainly composed of common fragments in ChemBL. A high SAscore is in principle associated with a molecule that should be difficult to synthesize. Figure 3 represents the distribution of these two scores for OD9_0 and OD9_1 (steps 1 and 3). It clearly appears that the molecules proposed by EvoMol are less ChemBL-like (with a CLscore lower than 3) and should be less synthesizable (with a with a SAscore distribution peak between 5 and 6). The search for new IFGs seems to lead to unusual associations between heteroatoms. Such unusual combinations are penalized by these scores.

**Fig. 4:**
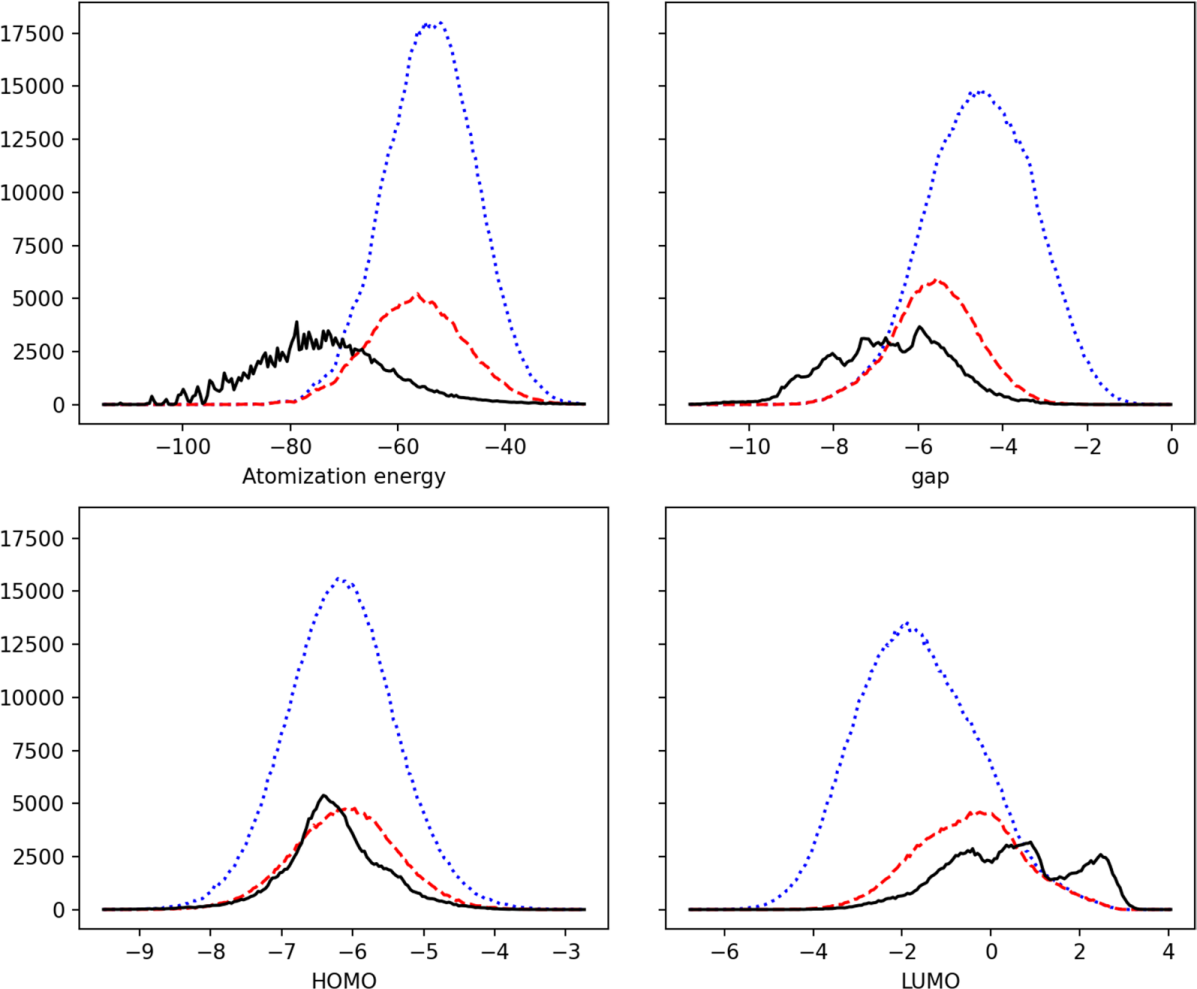
Distributions of the atomization energies (in hartree) and electronic energies (gap, HOMO and LUMO, in eV). The black straight lines represent the OD9_0 dataset (QM9UPC9). The new generated compounds OD9_1 are represented by the blue dotted lines for the step 2 or the red dashed lines for the step 3 of the workflow

A similar trend is observed for the electronic properties. Figure 4 represents the distribution of the atomization energy (total energy minus the sum of the atomic energies), the HOMO, LUMO and gap energies for OD9_0 and OD9_1. Here the steps 2 and 3 have been selected since step 1 precedes the DFT calculation. A large negative atomization energy is expected for very stable molecules. It is therefore comforting to be able to observe the strong similarity between this distribution and that of the SAscore. Concerning the energies of the frontier molecular orbitals, we can observe that the HOMO level is finally always centered around a value of about − 6 eV. In contrast to the very low energy of the LUMO (and therefore the small gap) for newly generated molecules. The new chemical diversity seems to correspond to very acceptor and unstable molecules. In the chemistry of molecular materials, many problems are related to electronic properties. A training dataset with much wider distributions of electronic properties should lead to more robust and relevant models for molecular materials.

**Fig. 5:**
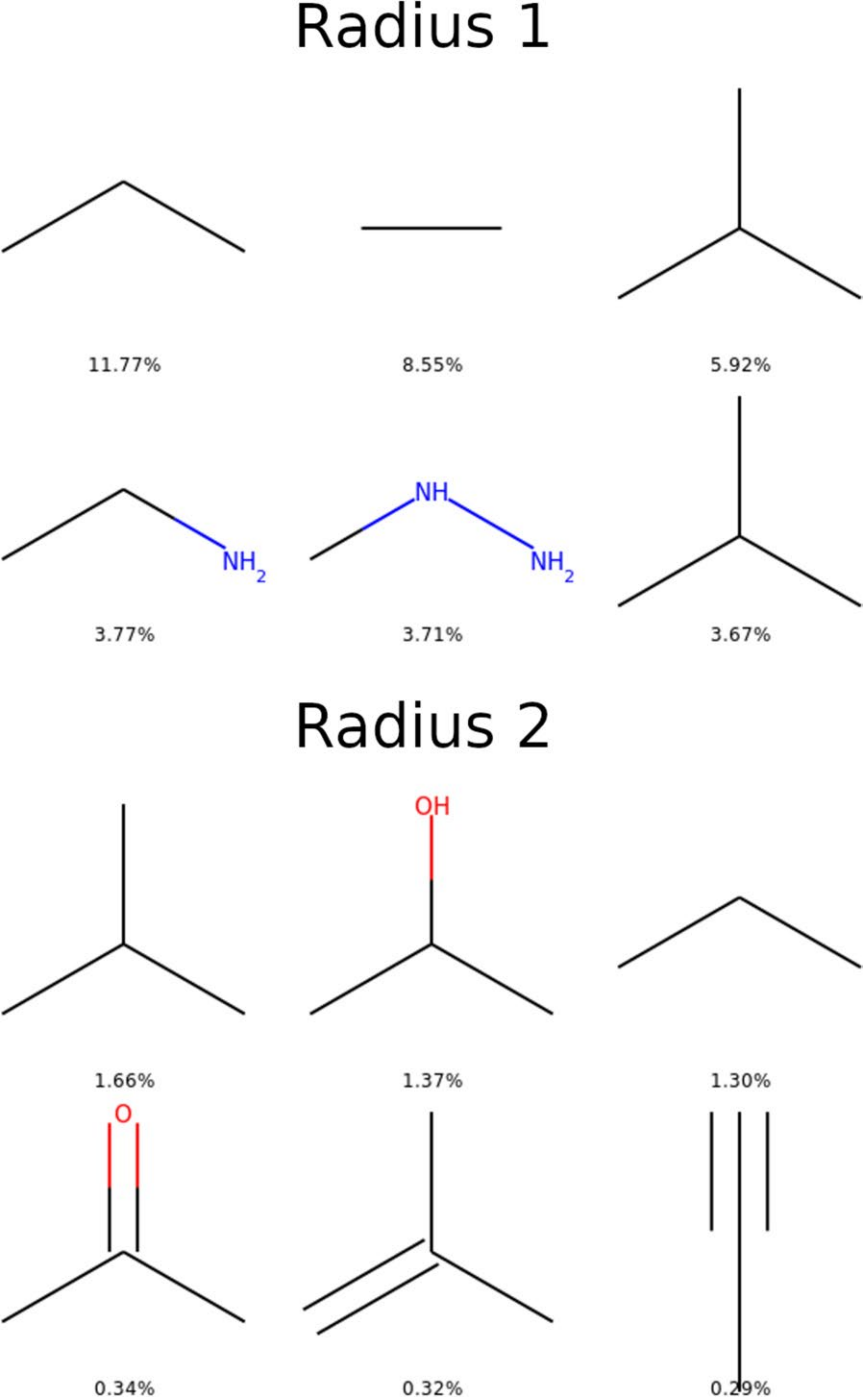
Top 3 molecular shingles with radius 1 (two first lines) and radius 2 (last two lines) for OD9_0 (up) and OD9_1 at step 3 (down)

Since the number of IFGs has skyrocketed during the generation, we decided to look at the shingles as descriptors. The diversity in terms of molecular shingles is reported in Table 2. The number of first neighbors configurations (radius of 1, denoted r1) is much lower in QM9 than in PC9. In fact, almost all QM9 shingles r1 exist in PC9 as revealed by the union of QM9 and PC9. However, when the radius of shingles increases, taking into account neighbors of neighbors (radius of 2) and so on (radius of 3), more chemical diversity in QM9 appears. QM9 is composed of less basic bricks than in PC9, but they are used in a combinatorial way, leading to what could be called a combinatorial diversity. EvoMol was able to propose 1007 shingles of radius 1 absent from QM9 and PC9 and an absurd amount of combinatorial diversity with 642,265 new shingles r2 and 4,568,964 new shingles r3. 80% of those are present only once (unique) in all the dataset. Just like for the IFGs, the drastic evolution of those numbers after DFT and after filtering, shows that exotic combinations of chemical environments are subject to common geometrical and electronic re-optimizations. They often cannot be written as Lewis structures, i.e. SMILES with a clear alternations between single and double bonds. The widespread SMILES representation can be an hindrance for de novo generation of complex electronic structures. Nevertheless, we managed to generate a lot of new molecular sub-graphs and combinations, stable in DFT. So much that it appears that r2 and r3 shingles are too specific to be used as descriptors to assess the molecular diversity. To observe redundancy in the data would then require huge data sets. In Fig. 5, the top three r1 and r2 shingles are represented along with the percentage of molecules presenting this descriptor. The top three are different in both datasets. Again we can observe that the percentage in OD9_1 (step 3) are lower, indicating a more evenly distributed distribution.

**Table 2:** Shingles statistics for the datasets at different stages of the workflow

Dataset	Size	Distinct shingles r1	Distinct shingles r2	Unique shingles r2	Distinct shingles r3	Unique shingles r3
QM9 step 3	122,227	229	28,053	7162	376,852	273,423
PC9 step 3	77,790	1295	39,725	18,718	223,127	158,226
OD9_0 step 3	184,158	1297	57,741	22,130	544,460	392,637
OD9_1 step 1	1,023,624	1007	642,265	282,311	4,568,964	3,675,203
OD9_1 step 2	854,059	3585	979,596	548,870	4,255,262	3,513,467
OD9_1 step 3	250,874	762	213,034	103,858	1,156,813	929,228
OD9 step 1	1,276,171	2447	691,715	301,669	5,156,545	4,064,788
OD9 step 2	1,088,773	3714	1,013,639	557,832	4,798,140	3,870,539
OD9 step 3	435,032	1563	250,163	116,483	1,665,725	1,293,995

A unique count represents a shingle that appears only once

**Fig. 6:**
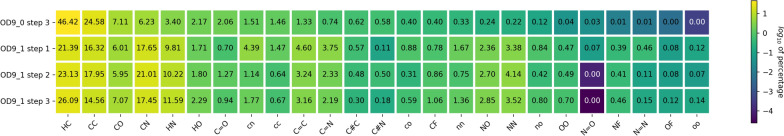
Annotated Heatmap representation of the distribution of the different chemical bonds. The percentage in each box is calculated on the basis of the number of such bonds in relation to the total number of bonds

Hoping to be able to better understand the chemical differences between OD9_0 and OD9_1, we decided to analyze their bond types and chemical functions according to CheckMol. Looking at a heatmap of the bonds in Fig. 6, we can see that CH and CC bonds represent a large majority of all the chemical bonds in OD9_0 (42.46% + 24.58%). Also, 15 of those 25 bonds are represented less than 1% including 6 bonds that are represented less than 0.05%. In the 1M SMILES generated with EvoMol, OD9_1 at step 1, there is in proportion far less CH and CC and much more CN and NH bonds. Many rare bonds concern two heteroatoms. We can see that using the IFG in our objective function was a great incentive for the generation of new examples of such bonds. There is still 12 bonds under 1% but no bond is under 0.05%. The DFT calculations and the step 3 of curation has a major impact on some bonds. Almost all N=O bonds and most N=N or aromatic CN bonds are discarded with our workflow.

**Fig. 7:**
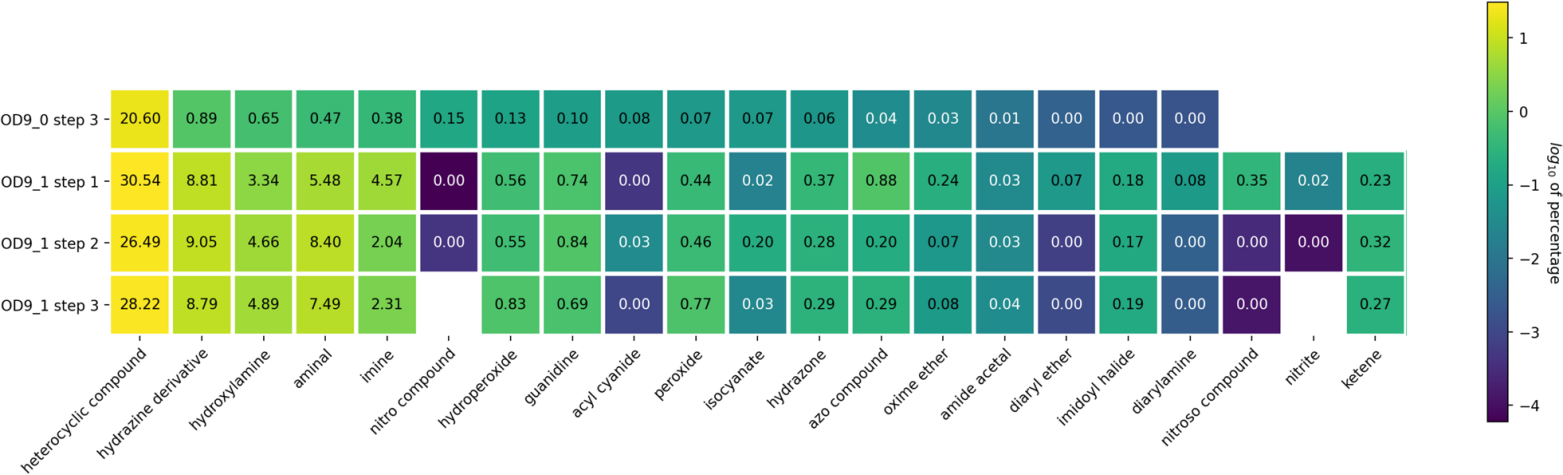
Annotated Heatmap representation of the distribution of a selection of functional groups detected by Checkmol. The percentage in each box is calculated on the basis of the number of such group in relation to the total number of functional groups

When we dig deeper into this study using functional groups (FGs) as detected by the Checkmol program, we found almost 100 distinct FGs. The table of all occurrences in all the datasets are given in the Additional file 2. We have selected FGs whose proportions show a strong evolution between the different datasets and represented their proportions in a heatmap (see Fig. 7). In all datasets, heterocyclic compounds are heavily represented and the use of the IFGs in our diversity objective has further amplified their proportion. We can then see that we have generated many hydrazine derivatives, hydroxylamines, aminals and imines. This is consistent with the significant increase in NN, CN, NH, C=N and NO bonds (see Fig. 6). Some rare functions have been generated quite often such as hydroperoxide, guanidine, peroxide, hydrazone, azo, oxime, diaryl ether, imidoyl halide, diarylamine. This partly justifies the increase in the proportion of oo (aromatic peroxyde), OO, C=N, CN, NN, N=N, NO, CF bonds. It can also be noted that three new FGs have appeared in OD9_1, nitroso, nitrite and ketene impacting the N=O, C=N and C=C bond ratios. On the contrary, EvoMol, cannot propose nitro compounds because in its actual stage, it does not handle formal charges and zwitterions. We can finally note that EvoMol has not increased the ratios of acyl cyanide or isocyanate compounds. The objective function based on scaffolds and IFGs allow for an interesting exploration of the chemistry of heteroatoms without guaranteeing an exhaustive exploration. Looking at the evolution of the ratios of these FGs, we can notice that the search for diversity has led us to generate rare chemical functions. However, in these small molecules with a limited number of atoms, the chemical functions are close to each other and prone to electronic reorganizations such as tautomeric equilibria.

### Case 2: goal-directed molecular generation

When the diversity and the QED are optimized jointly, improvement for both objectives are found. Table 3 reports the amounts of distinct descriptors in function of the type of optimized descriptor and the weight on the diversity term. A threshold effect can be observed on the QED experiment, beyond which increasing the weight on diversity does not produce more diversity in practice. But before this threshold, the diversity increases with the weight for all types of descriptors. Thus, it can be noticed that the descriptors are not independent. They follow the same trend. Optimizing the diversity of one type of descriptor increases the diversity of the other descriptors. When the entropy of the shingles is optimized with a high weight (e.g. 100 or 1000), the number of distinct shingles with radius 1 reaches more than 4600, while without entropy it only reaches 156. In the first case, a shingle is present on average in approximately 8 molecules whereas it is in 244 molecules in the second case. With high pressure on entropy, the chemical environments then become very singular and concentrate many heteroatoms. The impact is also visible on the scaffolds, with approximately 800 distinct scaffolds for 1000 molecules. The point where the weight on diversity is optimal is very unstable as we will see. The objective of QED is clearly antagonistic to that of diversity using such descriptors.

**Table 3:** Scaffolds, IFG and shingles statistics (averaged over 10 runs) for the QED goal-directed experiment for different descriptors and different weights for the entropy term

Optimized descriptor	Entropy weight	Mean QED	Distinct scaffolds	Distinct IFG	Distinct checkmol	Distinct shingles r1	Distinct shingles r2	Distinct shingles r3
None (i.e. QED only)	0	0.944	196	259	25	156	1854	5579
IFG	0.1	0.948	329	467	27	230	2570	6719
	1	0.947	670	859	44	375	4741	9627
	10	0.917	771	1221	63	684	6901	12,149
	100	0.048	648	2526	79	1302	12,902	20,799
	1000	0.034	607	2479	76	1314	12,714	20,586
Checkmol	0.1	0.948	265	375	32	191	2197	6141
	1	0.947	372	423	59	284	2985	7113
	10	0.925	415	470	106	365	3382	7596
	100	0.391	561	799	137	493	4950	11,006
	1000	0.074	545	929	140	604	5735	12,379
Shingles r1	0.1	0.948	466	600	38	451	3674	7765
	1	0.945	718	919	53	801	5978	10,423
	10	0.767	745	1176	76	2306	10,485	15,164
	100	0.036	798	1038	58	4681	23,328	30,813
	1000	0.036	802	1043	60	4802	23,305	30,803

**Fig. 8:**
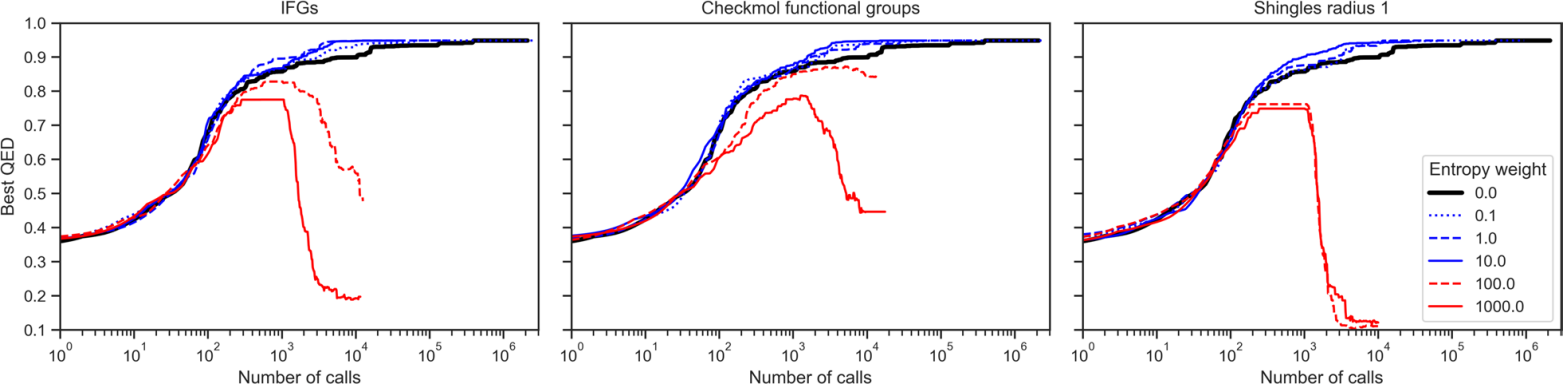
Evolution of the best solution for the QED goal-directed experiment (averaged over 10 runs) in function of the number of calls to the objective function for different descriptors and different weights for the entropy term

Figure 8 shows the evolution of the best QED value of the population compared to the number of calls to the evaluation function for different diversity parameters, i.e. type of descriptors and weights. On the one hand, with small but sufficient weight (blue curves) the generator converges faster towards a better solution. The scale being logarithmic, the improvement is of an order of magnitude, with no notable difference between the descriptors. The generator finds a compromise between intensification and exploration, so that it does not focus too quickly exclusively on the part of the chemical space that seems to be the most promising. On top of that, as dataset diversity increases over time, the solutions proposed are more diverse. In the end, when all the proposed solutions have good scores, the generator can continue to improve the dataset by offering more diversified solutions. On the other hand, when too much weight is put on diversity (red curves), the generator diverges to the point where even the best dataset solution declines over time. It is quite simple to understand that too much diversity will be counterproductive. In our experiments, the dataset is small, 1000 molecules, and the number of descriptors can be quite large as we have just seen. After some time, it is not enough to propose molecules with new descriptors, they must also be concentrated. If the weight is too important, it is the density of the descriptors that prevails over the initial objective.

**Fig. 9:**
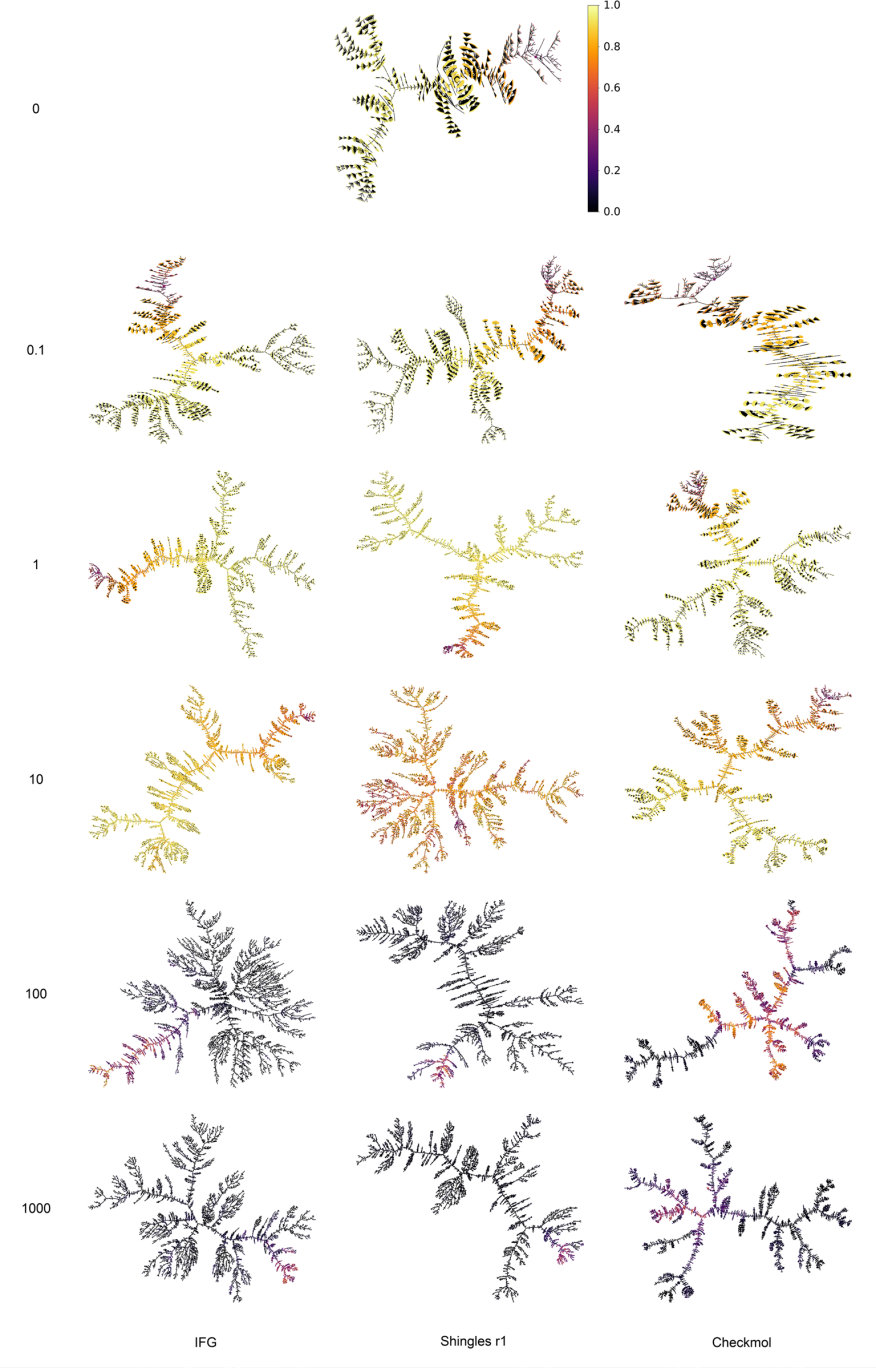
Exploration trees for the QED goal-directed experiment for different descriptors (in columns) and different weights for the entropy term (in rows). The color in the trees corresponds to the QED score only, i.e. without the diversity term

The exploration trees of Fig. 9 represent the relation of inheritance between successive individuals (edges) and the score of each individual (color). We present trees for different type of descriptors and different weights. The reference experiment, i.e. without entropy, will be used as a baseline for comparison. What is remarkable for this exploration tree is the low number of nodes and the very large number of direct descendants per node, materialized by the black triangles, which are juxtapositions of edges. This behavior is expected: without entropy, EvoMol will intensify around the most promising solutions, even if it means reducing the diversity of the population. This is the effect of the selection pressure of the evolutionary algorithm. For high weights, exploration trees are simple to interpret. Trees have many branches and are very scattered, i.e. few direct descendants per node. They quickly leave the area with good QEDs and intensively explore the descriptor space. The effect is a little less pronounced for the CheckMol functions because there are fewer of them. To increase diversity, the generator can no longer just discover new ones but must also concentrate as many as possible in each molecule. This strategy also ends up pushing the generator away from the good solutions for the QED objective. The evolution of the trees is more interesting when the weights on the entropy term are smaller. For descriptors such as shingles or IFG, with a low weight (0.1), the impact of entropy is already visible. Trees are more widespread and the number of direct descendants per node decreases. Areas with good solutions are also more developed. These effects are even more pronounced for a weight of 1. From a weight of 10, the trees take on a more orange hue as the solutions begin to deteriorate. We feel that exploration has taken precedence over intensification. For CheckMol functional groups, that are less numerous, the weight on the entropy must be slightly higher to see the same effects. This is due to the fact that the entropy term is not normalized and is therefore highly dependent on the number of descriptors and the size of the population.

## Conclusions

In this paper we present a fast and generic method to evaluate diversity according to descriptors that can be chosen or defined according to an application. Our innovative approach allows to individually estimate the impact of a solution to the diversity of a set, which allows for effective incremental evaluation. This metric can be used alone or in combination with other objectives. We have realized and made available an implementation in EvoMol, our molecular generator. This method can of course be easily incorporated into other generators based on population-based algorithms. It can be easily used to select a diverse subset of solutions from a large dataset, for active learning for instance. It is even possible to adapt it to force the diversity of deep learning molecular generators, as did Nigam et al. [36] to help his GAN model not fall into a *mode collapse* issue.

In terms of applications, we have shown two interesting results of adding a criterion of chemical diversity in de novo generation. Firstly, a diversity criterion based on scaffolds and chemical functions (IFGs for example) with a genetic algorithm has allowed to efficiently enhance the chemical diversity of reference datasets. Underrepresented chemical functions have been proposed. We have been able to study in detail the chemical diversity generated thanks to chemical functions, chemical bonds, shingles but also thanks to distributions of structural or electronic properties. For example, we observed an unprecedented exploration of the chemical space of acceptor molecules (low LUMO energy). The OD9 dataset with 435k molecules, thus represents an important improvement if the goal is to train a ML method with good performances in generalization.

Concerning the descriptors, we have probably gone through all the stable generic scaffolds in DFT. The same cannot be said for IFGs and shingles of radius 2 and 3 which are very often unique for molecules with 9 “heavy” atoms. This means that such descriptors are not generic enough. A more local approach seems mandatory. In this study, we manage to generate almost all the DFT stable shingles of radius 1, but encouraging a combinatorial diversity of these shingles could be interesting. Moreover, the learning on small molecules must be transferable to larger systems to be usable in practice. This local approach would be a step in this direction.

Secondly, we were able to measure the impact of diversity in objective-based generation problems. Getting a high QED score is not complicated, but adding a little diversity can cut the number of calls to the evaluation function by a factor of ten. Moreover, observation of the exploration trees shows that with this additional diversity criterion it is possible to further explore the chemical space. These results are very promising especially for the discovery of new molecular materials that rely on costly evaluation functions. However, it must be recognized that the amount of diversity must be limited and not exceed a threshold beyond which the diversity objective dominates over that of the property sought. This threshold will depend on each property. In addition, the method we propose depends on the chosen descriptors, therefore it is not an absolute diversity. It is obvious that some descriptors will be more or less adapted to a problem and in different proportions.

We assume that different structural-based descriptors should cover a wide range of problems. An immediate improvement would be to make the weights adjustment of the diversity automatic. We have shown through our goal-directed experiment that there is a breakpoint and therefore it would be possible to detect it automatically. We also think that we could use a method close to simulated annealing in order to vary these weights dynamically during the search. Although we can automatically adjust the weights, in some cases expert knowledge may be required to choose or design problem-specific descriptors.

Another room for improvement is that under certain conditions, the criterion of diversity leads to the concentration of too many descriptors in each solution. Quite simply, it would be possible to add a penalization term that would depend on the number of descriptors or to replace the sum by a maximum in the calculation of the individual contribution (Eqs. 6 and 8).

Finally, the results of this article show that of all the molecular graphs that can be proposed, a large number are problematic when written in the form of SMILES (i.e. Lewis structures) or when calculated in DFT. In order to improve the generation of molecules, one could establish which descriptors would be relevant to discriminate stable molecules from unstable ones. Lists of forbidden fragments or combinations would allow to limit the amount of calculations in quantum chemistry. The coupling of synthesizability scores could also be promising.

## Supplementary Information

**Additional file 1.** Additional tables.

**Additional file 2.** Detailed CheckMol analysis.

## Data Availability

The EvoMol code source is available in the following github repository:https://github.com/jules-leguy/EvoMol. The OD9 dataset is available as a figshare collection. It encompasses the 435 032 NWChem logfiles calculated thanks to the QuChemPedIA@home BOINC project.10.6084/m9.figshare.c.5180513.v1.
