# Supplementary material for: Scalable estimator of the diversity for de novo molecular generation resulting in a more robust QM dataset (OD9) and a more efficient molecular optimization
Source: J Cheminform. 2021 Oct 2;13:76. doi: 10.1186/s13321-021-00554-8 (PMC8487551; doi:10.1186/s13321-021-00554-8)
Supplement: Supplementary file 1 — Additional file 1. Additional tables. [file 13321_2021_554_MOESM1_ESM.pdf]

RESEARCH

Scalable estimator of the diversity for de novo molecular generation resulting in a more robust QM dataset (OD9) and a more efficient molecular optimization.

Jules Leguy, Marta Glavatskikh, Thomas Cauchy and Benoit Da Mota

1 Additional Tables

Full list of author information is available at the end of the article

Table 1 Evolution of the number of molecules passing the tests of the workflow.

	QM9			PC9			OD9_1		
**Step**	**True**	**False**	**% False**	**True**	**False**	**% False**	**True**	**False**	**% False**
1. BOINC submissions	133 885			118 662			1 023 624		
2.a Completed calculations	133 069	816	0.61	113 036	5 626	4.74	957 178	66 447	6.49
2.b Validated calculations	131 601	1 468	1.10	109 096	3 940	3.49	907 624	49 554	5.18
2.c No NaN / Has_orig_smi / Has_smi	131 380	221	0.17	107 820	1 276	1.17	907 624	0	0
2.d Canonical SMILES after DFT	128 958	2 422	1.84	105 756	2 064	1.91	854 059	53 565	5.9
3.a Same SMILES before and after DFT	122 307	6 651	5.16	96 839	8 917	8.43	250 874	603 185	70.63
3.b Not Duplicated	122 227	80	0.07	77 790	19 049	19.67	250 874	0	0
Workflow's passing rate (%)	91.29			65.56			24.51		

Table 2 Scaffolds and IFG statistics at different stages of the workflow for the datasets.

			Generic scaffolds		Scaffolds			IFG	
**Dataset**	**Step**	**Size**	**Distinct**	**Unique**	**Distinct**	**Unique**	**Acyclic**	**Distinct**	**Unique**
QM9	1	133 885	1 965	0	15 989	12 547	13 998	7 925	5 770
	2	128 958	2 966	277	15 747	12 343	13 291	8 528	6 238
	3	122 227	1 964	7	14 060	11 019	12 615	6 981	5 097
PC9	1	118 662	4 702	1 847	8 437	5 810	48 242	17 870	12 724
	2	105 756	3 241	1 187	7 421	5 006	43 402	16 270	11 576
	3	77 790	2 772	1 108	6 566	4 708	31 542	13 887	10 351
OD9_0	1	252 547	5 713	1 705	22 438	16 227	62 240	24 928	17 855
	2	234 714	4 923	1 146	21 217	15 360	56 693	23 693	16 999
	3	184 158	3 798	959	18 850	14 531	40 103	20 075	15 053
OD9_1	1	1 023 624	9 163	900	460 978	371 545	28 725	461 247	387 483
	2	854 059	108 832	46 853	334 256	264 881	66 078	428 136	365 682
	3	250 874	4 858	469	88 094	66 735	15 956	124 396	104 685
OD9	1	1 276 171	12 929	2 463	480 464	385 757	90 965	482 009	402 847
	2	1 088 773	109 573	46 852	351 845	277 722	122 771	446 367	378 905
	3	435 032	6 776	1 252	104 529	79 643	56 059	141 090	117 495

Table 3 Shingles (with different radii) statistics at different stages of the workflow for the datasets.

			Shingles					
**Dataset**	**Step**	**Size**	**Distinct r 1**	**Unique r 1**	**Distinct r 2**	**Unique r 2**	**Distinct r 3**	**Unique r 3**
QM9	1	133 885	462	66	33 818	9 419	419 384	305 153
	2	128 958	641	83	38 205	12 602	410 882	300 861
	3	122 227	229	2	28 053	7 162	376 852	273 423
PC9	1	118 662	1 726	321	55 641	26 065	284 341	186 072
	2	105 756	1 471	276	46 299	21 146	250 603	161 835
	3	77 790	1 295	258	39 725	18 718	223 127	158 226
OD9_0	1	252 547	1 958	379	78 564	31 330	642 717	431 386
	2	234 714	1 511	262	71 562	28 008	600 939	403 923
	3	184 158	1 297	255	57 741	22 130	544 460	392 637
OD9_1	1	1 023 624	1 007	25	642 265	282 311	4 568 964	3 675 203
	2	854 059	3 585	364	979 596	548 870	4 255 262	3 513 467
	3	250 874	762	30	213 034	103 858	1 156 813	929 228
OD9	1	1 276 171	2 447	362	691 715	301 669	5 156 545	4 064 788
	2	1 088 773	3 714	393	1 013 639	557 832	4 798 140	3 870 539
	3	435 032	1 563	244	250 163	116 483	1 665 725	1 293 995

Table 4 Bonds statistics for the datasets at different stages of the workflow. The percentage correspond to the bond counts compared to the total number of bonds.

	**OD9_0 at step 3**		**OD9_1 step 1**		**OD9_1 step 2**		**OD9_1 step 3**	
**Bond**	**count**	**%**	**count**	**%**	**count**	**%**	**count**	**%**
HC	1 582 896	46.42	3 341 732	21.39	2 927 412	23.13	1 052 581	26.09
CC	838 253	24.58	2 550 406	16.32	2 271 741	17.95	587 407	14.56
CO	242 418	7.11	938 841	6.01	752 491	5.95	285 118	7.07
CN	212 293	6.23	2 758 014	17.65	2 658 599	21.01	703 800	17.45
HN	115 841	3.40	1 532 522	9.81	1 293 642	10.22	467 596	11 59
HO	73 860	2.17	267 216	1.71	228 189	1.80	92 208	2.29
C=O	70 091	2.06	108 597	0.70	160 574	1.27	37 883	0.94
cn	51 619	1.51	686 183	4.39	144 598	1.14	71 374	1.77
cc	49 769	1.46	230 381	1.47	81 365	0.64	26 977	0.67
C=C	45 304	1.33	719 432	4.60	410 562	3.24	127 494	3.16
C=N	25 363	0.74	586 210	3.75	294 593	2.33	88 244	2.19
C#C	20 989	0.62	88 635	0.57	60 224	0.48	11 954	0.30
C#N	19 736	0.58	17 743	0.11	63 836	0.50	7 079	0.18
co	13 580	0.40	137 877	0.88	39 833	0.31	23 792	0.59
CF	13 558	0.40	122 423	0.78	108 222	0.86	42 818	1.06
nn	11 168	0.33	260 549	1.67	94 776	0.75	54 698	1.36
NO	8 267	0.24	368 102	2.36	341 288	2.70	115 085	2.85
NN	7 501	0.22	528 736	3.38	523 442	4.14	142 015	3.52
no	4 182	0.12	131 695	0.84	53 329	0.42	32 338	0.80
OO	1 528	0.04	73 012	0.47	61 699	0.49	28 146	0.70
N=O	1 168	0.03	11 698	0.07	13	0.00	1	0.00
NF	444	0.01	60 793	0.39	51 880	0.41	18 749	0.46
N=N	298	0.01	72 313	0.46	14 006	0.11	6 018	0.15
OF	161	0.00	12 583	0.08	9 895	0.08	4 719	0.12
oo	15	0.00	19 386	0.12	8 923	0.07	5 818	0.14
